# Transcatheter aortic valve implantation amid the COVID-19 pandemic: a nationwide analysis of the first COVID-19 wave in the Netherlands

**DOI:** 10.1007/s12471-022-01704-9

**Published:** 2022-06-01

**Authors:** M. J. P. Rooijakkers, W. W. L. Li, N. A. Stens, M. M. Vis, P. A. L. Tonino, L. Timmers, N. M. Van Mieghem, P. den Heijer, S. Kats, P. R. Stella, V. Roolvink, H. W. van der Werf, M. G. Stoel, C. E. Schotborgh, G. Amoroso, F. Porta, F. van der Kley, M. H. van Wely, H. Gehlmann, L. A. F. M. van Garsse, G. S. C. Geuzebroek, M. W. A. Verkroost, J. M. Mourisse, N. M. Medendorp, N. van Royen

**Affiliations:** 1 Department of Cardiology, Radboud University Medical Centre, Nijmegen, The Netherlands; 2 Department of Cardiothoracic Surgery, Radboud University Medical Centre, Nijmegen, The Netherlands; 3 Department of Physiology, Radboud University Medical Centre, Nijmegen, The Netherlands; 4 Department of Cardiology, Amsterdam University Medical Centre, Amsterdam, The Netherlands; 5 Department of Cardiology, Catharina Hospital, Eindhoven, The Netherlands; 6 Department of Cardiology, St. Antonius Hospital, Nieuwegein, The Netherlands; 7 Department of Cardiology, Erasmus University Medical Centre, Rotterdam, The Netherlands; 8 Department of Cardiology, Amphia Hospital, Breda, The Netherlands; 9 Department of Cardiothoracic Surgery, Maastricht University Medical Centre, Maastricht, The Netherlands; 10 Department of Cardiology, University Medical Centre Utrecht, Utrecht, The Netherlands; 11 Department of Cardiology, Isala Hospital, Zwolle, The Netherlands; 12 Department of Cardiology, University Medical Centre Groningen, Groningen, The Netherlands; 13 Department of Cardiology, Medisch Spectrum Twente, Enschede, The Netherlands; 14 Department of Cardiology, Haga Hospital, The Hague, The Netherlands; 15 Department of Cardiology, OLVG Hospital, Amsterdam, The Netherlands; 16 Department of Cardiothoracic Surgery, Leeuwarden Medical Centre, Leeuwarden, The Netherlands; 17 Department of Cardiology, Leiden University Medical Centre, Leiden, The Netherlands; 18 Department of Anaesthesiology, Pain and Palliative Medicine, Radboud University Medical Centre, Nijmegen, The Netherlands; 19 Netherlands Heart Registration, Utrecht, The Netherlands

**Keywords:** Aortic valve stenosis, COVID-19, Postoperative complications, Registries, Transcatheter aortic valve implantation, Treatment outcome

## Abstract

**Introduction:**

The coronavirus disease 2019 (COVID-19) pandemic has put tremendous pressure on healthcare systems. Most transcatheter aortic valve implantation (TAVI) centres have adopted different triage systems and procedural strategies to serve highest-risk patients first and to minimise the burden on hospital logistics and personnel. We therefore assessed the impact of the COVID-19 pandemic on patient selection, type of anaesthesia and outcomes after TAVI.

**Methods:**

We used data from the Netherlands Heart Registration to examine all patients who underwent TAVI between March 2020 and July 2020 (COVID cohort), and between March 2019 and July 2019 (pre-COVID cohort). We compared patient characteristics, procedural characteristics and clinical outcomes.

**Results:**

We examined 2131 patients who underwent TAVI (1020 patients in COVID cohort, 1111 patients in pre-COVID cohort). EuroSCORE II was comparable between cohorts (COVID 4.5 ± 4.0 vs pre-COVID 4.6 ± 4.2, *p* = 0.356). The number of TAVI procedures under general anaesthesia was lower in the COVID cohort (35.2% vs 46.5%, *p* < 0.001). Incidences of stroke (COVID 2.7% vs pre-COVID 1.7%, *p* = 0.134), major vascular complications (2.3% vs 3.4%, *p* = 0.170) and permanent pacemaker implantation (10.0% vs 9.4%, *p* = 0.634) did not differ between cohorts. Thirty-day and 150-day mortality were comparable (2.8% vs 2.2%, *p* = 0.359 and 5.2% vs 5.2%, *p* = 0.993, respectively).

**Conclusions:**

During the COVID-19 pandemic, patient characteristics and outcomes after TAVI were not different than before the pandemic. This highlights the fact that TAVI procedures can be safely performed during the COVID-19 pandemic, without an increased risk of complications or mortality.

**Supplementary Information:**

The online version of this article (10.1007/s12471-022-01704-9) contains supplementary material, which is available to authorized users.

## What’s new?

- The clinical characteristics of patients undergoing transcatheter aortic valve implantation (TAVI) during the COVID-19 pandemic (COVID cohort) were comparable to those of patients undergoing TAVI during the pre-COVID-19 era (pre-COVID cohort).
- The percentage of TAVI procedures performed using general anaesthesia was lower in the COVID cohort, with more transaxillary and fewer transapical TAVIs in the COVID cohort compared to the pre-COVID cohort.
- TAVI procedures can be safely performed amid the COVID-19 pandemic, without an increased risk of complications or mortality.
- The COVID-19 pandemic might have accelerated the shift towards more TAVI procedures with local anaesthesia/conscious sedation, a trend which was already observed before this ongoing pandemic.

## Introduction

The coronavirus disease 2019 (COVID-19) pandemic has put tremendous pressure on healthcare systems worldwide, leading to the deferment of large numbers of elective procedures [1, 2]. Patients with symptomatic severe aortic stenosis (AS) have a poor prognosis if left untreated, with an annual mortality of approximately 50% [3].

Transcatheter aortic valve implantation (TAVI) is an established, minimally invasive treatment option in patients with severe AS, a condition which is found in a predominantly elderly, vulnerable population with serious comorbidities. Postponement of this potentially life-saving procedure is associated with a risk of sudden cardiac death or irreversible cardiac deterioration. Previous literature reported a ‘waiting list’ mortality rate of 23.3% and 27.5% at 6 and 12 months, respectively, in patients awaiting TAVI [4]. Data from the UK TAVI Registry demonstrated a significant decrease in TAVI activity following the COVID-19 outbreak with an estimated 5000 patients with severe AS not receiving treatment (either surgical aortic valve replacement (SAVR) or TAVI) during the period March to November 2020 [5].

Multiple position statements have been published on when to perform TAVI for symptomatic severe AS amid the COVID-19 crisis [6–9]. However, the risks of adverse events caused by postponement of these interventions should be balanced against the risks of contracting COVID-19 when hospitalised for this high-risk cardiovascular intervention [10]. Also, most TAVI centres have adopted different triage systems and different procedural strategies during the COVID-19 pandemic, aiming to serve highest-risk patients first and to minimise the burden on hospital logistics and personnel [11].

We previously reported on the safety and feasibility of a continued TAVI programme during the COVID-19 pandemic in our own centre [10]. Here we present data from the Netherlands Heart Registration (NHR), a nationwide registry in which data on all cardiac interventions performed in the Netherlands are collected. We studied whether the implementation of a different triage system during the COVID-19 pandemic led to a different ‘case mix’ of TAVI patients. We also assessed whether the COVID-19 pandemic, with its associated reduction in the number of available intensive care unit (ICU) beds and anaesthesiologists, has led to a shift from general anaesthesia to local anaesthesia. Finally, we studied whether the outcomes in terms of complications and mortality after TAVI were different during the COVID-19 pandemic as compared to the pre-COVID-19 era.

## Methods

### Data collection

In the NHR, data on all cardiac interventions performed in the Netherlands are registered by all Dutch heart centres. Data are reported per site and collected on a yearly basis. During the study period, 15 of these hospitals performed TAVI procedures (Table S1, Electronic Supplementary Material). Data are collected on patient characteristics, procedural characteristics and outcome variables [12]. Data collection and registration are performed by the participating centres in a secure online environment. A waiver of consent for the NHR data registration was obtained from the Medical Ethics Committee. The study was conducted in accordance with the guiding principles of the Declaration of Helsinki. In order to obtain data for research purposes, the data request must be reviewed and approved by the NHR [13]. Only data necessary to answer the prespecified research questions are provided. To prevent identification of centre-specific data, requested variables are provided for all TAVI centres together.

### Population and design

In this nationwide, retrospective multicentre study, we examined all patients who underwent a TAVI procedure for symptomatic severe AS between March 2020 and July 2020 (COVID cohort), and between March 2019 and July 2019 (pre-COVID cohort). We specifically selected these 5‑month periods since the period from March 2020 to July 2020 comprised the first COVID-19 wave in the Netherlands [14]. The maximum follow-up duration of 150 days was determined by the period between final data extraction and the date of the TAVI procedure of the last patient included in the COVID cohort.

### Procedure

All centres performed TAVI according to their respective local protocols. Type of TAVI device, valve size and TAVI access were left to the operator’s discretion. The TAVI procedure was performed using general anaesthesia or local anaesthesia with or without conscious sedation.

### Endpoints

The primary endpoint was mortality at 150 days. Secondary endpoints included procedural mortality (defined as mortality within 72 h after the intervention), mortality at 30 days, stroke during hospitalisation, major vascular complications according to Valve Academic Research Consortium (VARC) criteria [15], permanent pacemaker implantation and aortic valve reintervention within 150 days.

### Statistical analysis

Categorical variables are presented as number with percentage. Continuous variables are presented as mean with standard deviation or median with interquartile range (IQR), depending on distribution. Categorical variables were compared using the chi-square or Fisher’s exact test, as appropriate. Continuous variables were compared using Student’s *t*-test or Mann-Whitney U test, as appropriate. Survival was estimated by means of Kaplan-Meier analysis and was compared with the use of the log-rank test. A *p*-value of < 0.05 was considered statistically significant. Analyses were performed using IBM SPSS Statistics software version 26.0 (IBM Corp., Armonk, NY, USA).

## Results

### Baseline characteristics

We examined a total of 2131 patients, of whom 1020 underwent a TAVI procedure between March 2020 and July 2020 (COVID cohort) and 1111 underwent a TAVI procedure between March 2019 and July 2019 (pre-COVID cohort). Of the 1020 patients treated with TAVI in the COVID cohort, 38 (3.7%) were initially planned for SAVR. Baseline characteristics are shown in Tab. 1. Mean age was 79.2 ± 6.9 years and 54.2% of patients were men. The majority of baseline characteristics did not differ between the two time periods, but exceptions were previous aortic valve surgery (COVID 5.1% vs pre-COVID 3.2%, *p* = 0.032), extracardiac arterial pathology (15.7% vs 19.6%, *p* = 0.019), previous stroke (13.2% vs 10.2%, *p* = 0.029) and left ventricular ejection fraction (49.8% vs 50.8%, *p* = 0.028). EuroSCORE II was comparable (COVID 4.5 ± 4.0 vs pre-COVID 4.6 ± 4.2, *p* = 0.356). New York Heart Association class was lower in the COVID cohort, whereas the proportion of emergent TAVI procedures was higher in the COVID cohort (COVID 0.6% vs pre-COVID 0%, *p* = 0.012).

**Table 1:** Baseline characteristics

	Total cohort (*n* = 2131)	Pre-COVID (*n* = 1111)	COVID (*n* = 1020)	*p*-value
*Demographics*				
Age, years	79.2 ± 6.9	79.3 ± 7.0	79.1 ± 6.7	0.483
Male sex, *n* (%)	1155 (54.2)	609 (54.8)	546 (53.5)	0.552
Height, cm	169.5 ± 9.3	169.3 ± 9.3	169.8 ± 9.2	0.187
Weight, kg	78.1 ± 15.6	77.6 ± 15.5	78.8 ± 15.7	0.077
Body mass index (BMI), kg/m^2^	27.1 ± 5.0	27.0 ± 5.0	27.3 ± 4.9	0.249
– Obesity (BMI > 30), *n* (%)	509 (24.0)	266 (24.0)	243 (23.9)	0.961
*Medical history*				
Diabetes mellitus on insulin, *n* (%)	189 (8.9)	90 (8.1)	99 (9.7)	0.193
Recent myocardial infarction (< 90 days prior to TAVI), *n* (%)	36 (1.7)	17 (1.5)	19 (1.9)	0.553
Previous cardiac surgery, *n* (%)	362 (17.0)	195 (17.6)	167 (16.4)	0.458
Previous aortic valve surgery, *n* (%)	88 (4.1)	36 (3.2)	52 (5.1)	0.032
Extracardiac arterial pathology, *n* (%)	377 (17.7)	217 (19.6)	160 (15.7)	0.019
Previous stroke, *n* (%)	248 (11.6)	113 (10.2)	135 (13.2)	0.029
Serum creatinine, µmol/l	102.7 ± 56.6	102.5 ± 50.1	103.0 ± 63.0	0.840
Dialysis, *n* (%)	18 (0.8)	8 (0.7)	10 (1.0)	0.513
Chronic pulmonary disease, *n* (%)	370 (17.4)	197 (17.8)	173 (17.0)	0.633
Pulmonary artery pressure, mm Hg	29.1 ± 8.9	28.9 ± 8.7	29.2 ± 9.1	0.531
Prior pacemaker, *n* (%)	183 (8.6)	95 (8.6)	88 (8.6)	0.960
Neurological dysfunction, *n* (%)	93 (4.4)	40 (3.6)	53 (5.2)	0.076
Poor mobility, *n* (%)	187 (8.8)	100 (9.0)	87 (8.6)	0.692
*NYHA class*				
– Class I, *n* (%)	245 (11.8)	127 (11.8)	118 (11.8)	0.979
– Class II, *n* (%)	681 (32.8)	317 (29.5)	364 (36.3)	0.001
– Class III, *n* (%)	1017 (49.0)	560 (52.1)	457 (45.6)	0.003
– Class IV, *n* (%)	134 (6.5)	71 (6.6)	63 (6.3)	0.769
Critical preoperative state, *n* (%)	7 (0.3)	4 (0.4)	3 (0.3)	1.000
*Urgency of procedure*				
– Elective, *n* (%)	1880 (88.8)	994 (89.5)	886 (87.9)	0.229
– Urgent, *n* (%)	232 (11.0)	116 (10.5)	116 (11.5)	0.436
– Emergent, *n* (%)	6 (0.3)	0	6 (0.6)	0.012
– Salvage, *n* (%)	0	0	0	N/A
LVEF, %	50.3 ± 10.0	50.8 ± 9.7	49.8 ± 10.3	0.028
Right bundle branch block,* n* (%)	150 (8.5)	88 (9.6)	62 (7.3)	0.079
EuroSCORE II	4.6 ± 4.1	4.6 ± 4.2	4.5 ± 4.0	0.356

Data are presented as mean ± standard deviation or as number (%)

*COVID* coronavirus disease 2019, *EuroSCORE* European System for Cardiac Operative Risk Evaluation, *NYHA* New York Heart Association, *LVEF* left ventricular ejection fraction, *TAVI* transcatheter aortic valve implantation

### Procedural characteristics

**Fig. 1:**
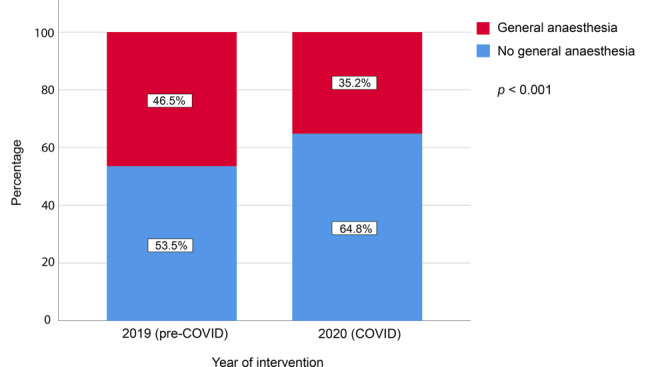
Type of anaesthesia used

Details about procedural characteristics are provided in Tab. 2. A total of 875 (41.1%) TAVI procedures were performed using general anaesthesia, with a significantly lower proportion in the COVID cohort as compared to the pre-COVID cohort (35.2% vs 46.5%, *p* < 0.001) (Fig. 1). The transfemoral approach was predominantly used (85.8%), followed by the transaxillary (6.0%), direct transaortic (4.9%) and transapical (3.2%) approaches. The transaxillary approach was used more frequently in the COVID cohort (7.8% vs 4.4%, *p* = 0.001) and the transapical approach less frequently (2.2% vs 4.1%, *p* = 0.013) when compared to the pre-COVID cohort.

**Table 2:** Procedural characteristics

	Total cohort (*n* = 2131)	Pre-COVID (*n* = 1111)	COVID (*n* = 1020)	*p*-value
General anaesthesia, *n* (%)	875 (41.1)	516 (46.5)	359 (35.2)	< 0.001
*TAVI access site*				
– Transfemoral, *n* (%)	1821 (85.8)	959 (86.4)	862 (85.2)	0.422
– Transaxillary, *n* (%)	128 (6.0)	49 (4.4)	79 (7.8)	0.001
– Transapical, *n* (%)	67 (3.2)	45 (4.1)	22 (2.2)	0.013
– Direct transaortic, *n* (%)	104 (4.9)	56 (5.0)	48 (4.7)	0.748
– Other, *n* (%)	2 (0.1)	1 (0.1)	1 (0.1)	1.000
Predilation, *n* (%)	766 (36.2)	371 (33.8)	395 (38.8)	0.016
Postdilation, *n* (%)	306 (14.5)	159 (14.5)	147 (14.5)	0.956

Data are presented as number (%)

*COVID* coronavirus disease 2019, *TAVI* transcatheter aortic valve implantation

### Clinical outcomes

**Fig. 2:**
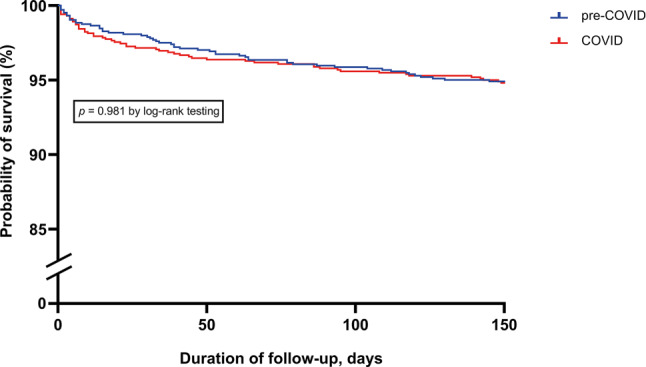
Kaplan-Meier survival curves

Clinical outcomes are presented in Tab. 3. Median duration of hospitalisation was 4 (IQR 3–7) days, which was comparable between the two groups. The incidences of stroke during hospitalisation (COVID 2.7% vs pre-COVID 1.7%, *p* = 0.134), major vascular complications within 30 days (2.3% vs 3.4%, *p* = 0.170), permanent pacemaker implantation (10.0% vs 9.4%, *p* = 0.634), aortic valve reintervention within 30 days (0.2% vs 0.3%, *p* = 1.000) and aortic valve reintervention within 150 days (0.5% vs 0.7%, *p* = 0.754) did not differ significantly between the two time periods. Procedural mortality (COVID 0.9% vs pre-COVID 1.0%, *p* = 0.853), 30-day mortality (2.8% vs 2.2%, *p* = 0.359) and 150-day mortality (5.2% vs 5.2%, *p* = 0.993) were comparable between the two groups. Fig. 2 depicts the Kaplan-Meier survival curves.

**Table 3:** Clinical outcomes

	Total cohort (*n* = 2131)	Pre-COVID (*n* = 1111)	COVID (*n* = 1020)	*p*-value
Duration of hospitalisation, days	4 (3–7)	5 (3–7)	4 (3–7)	0.068
Stroke during hospitalisation, *n* (%)	46 (2.2)	19 (1.7)	27 (2.7)	0.134
Major vascular complication < 30 days, *n* (%)	51 (2.9)	30 (3.4)	21 (2.3)	0.170
Permanent pacemaker implantation, *n* (%)	195 (9.7)	94 (9.4)	101 (10.0)	0.634
Aortic valve reintervention < 30 days, *n* (%)	5 (0.3)	3 (0.3)	2 (0.2)	1.000
Aortic valve reintervention < 150 days, *n* (%)	10 (0.6)	6 (0.7)	4 (0.5)	0.754
Procedural mortality,* n* (%)	19 (0.9)	10 (1.0)	9 (0.9)	0.853
30-day mortality, *n* (%)	52 (2.5)	23 (2.2)	29 (2.8)	0.359
150-day mortality, *n* (%)	107 (5.2)	54 (5.2)	53 (5.2)	0.993

Data are presented as median (interquartile range) or as number (%)

*COVID* coronavirus disease 2019

## Discussion

In this nationwide cohort study, we compared the patient characteristics, procedural characteristics and clinical outcomes of a patient cohort undergoing TAVI amid the COVID-19 pandemic to a cohort undergoing TAVI in the same time period a year before (pre-COVID-19).

The key findings can be summarised as follows. First, regarding case mix changes, mean EuroSCORE II was similar (COVID 4.5 vs pre-COVID 4.6) in both cohorts. Second, the percentage of TAVI procedures performed using general anaesthesia was lower in the COVID cohort (35.2% vs 46.5%), with more transaxillary and fewer transapical TAVIs in the COVID cohort compared to the pre-COVID cohort (7.8% vs 4.4% and 2.2% vs 4.1%, respectively). Third, the 30-day and 150-day mortality in the two cohorts was comparable (COVID 2.8% and 5.2% vs pre-COVID 2.2% and 5.2%, respectively) with a comparable complication rate (i.e. stroke, major vascular complication, permanent pacemaker implantation and aortic valve reintervention).

Our data support the hypothesis that TAVI procedures can be safely performed amid this global crisis, with mortality and complication rates comparable to the pre-COVID era. These findings are consistent with previous research reporting on the safety and feasibility of a continued TAVI programme during the COVID-19 pandemic, without an increased risk of complications or mortality [16, 17].

The EuroSCORE II, a risk score used to predict 30-day mortality, did not differ between the two time periods. This indicates that, although a different triage system was adopted by most centres in the Netherlands at the height of the COVID-19 pandemic, factors other than the EuroSCORE (e.g. patient symptomatology and echocardiographic features) are used for triage.

During the COVID-19 pandemic, general anaesthesia has been used less frequently than during the reference period. A plausible explanation might be that the ICU capacity was mainly reserved for COVID-19 patients needing endotracheal intubation and ventilation. In addition, as a result of the established safety of local anaesthesia protocols in the literature [18], the increased adoption of local anaesthesia/conscious sedation may also have been observed independent of this pandemic. The majority of TAVI procedures are performed using a transfemoral approach, which facilitates the use of local anaesthesia/conscious sedation instead of general anaesthesia. The increased use of local anaesthesia/conscious sedation was not accompanied by an increase in adverse clinical outcomes, which is in line with previous reports [18, 19].

Some centres have introduced a nurse-led TAVI approach in selected transfemoral TAVI patients, in which the catheterisation laboratory nurse administers local anaesthesia [11]. In order to further reduce the burden on hospital logistics and personnel, a same-day discharge approach can be used in carefully selected patients following uncomplicated TAVI, achieving similar 30-day outcomes to those with a next-day discharge [20].

It might be expected that some surgical candidates would be treated with TAVI instead of SAVR, resulting in a larger decrease in SAVRs than in TAVIs in the COVID cohort. However, this is not supported by the relatively low number of TAVI patients (3.7%) that were initially planned for SAVR. Also, in the NHR handbook published online, the total numbers of TAVIs and SAVRs performed in 2019 and 2020 are provided [21].

The total numbers of TAVI procedures in 2019 and 2020 were 2791 and 2539, respectively, showing a decrease of 9.0%. Regarding SAVR, the respective total numbers in 2019 and 2020 were 1365 and 1268, corresponding to a decrease of 7.1%. Thus, the percentage decrease in the total number of procedures performed in 2020 (COVID year) was not higher for SAVR than for TAVI.

### Limitations

First, all data were collected in the NHR. The Dutch Society of Cardiology and Dutch Society of Cardiothoracic Surgery determine on an annual basis which variables must be provided by TAVI centres, thus limiting the data we can provide in the present study. The number of mandatory outcome variables is relatively small, and therefore some VARC-3-related endpoints are missing (e.g. data on bleeding complications and rehospitalisation) [15]. Second, we only have cumulative data rather than data specifically related to individual TAVI centres. Therefore, inter-hospital differences could not be assessed. Third, since we only had access to data regarding TAVI, data regarding SAVR are lacking in our analysis. Finally, data on long-term follow-up were not yet available for the present analysis.

### Future perspectives

Although the number of COVID-19 vaccine doses administered globally is as high as 11.3 billion, and the number of fully vaccinated persons has passed 4.5 billion [22], there is a continuous risk of (re)infection. In the case of increasing COVID-19 admissions, the strain on healthcare systems can potentially be diminished by using a same-day discharge approach in selected TAVI patients [20]. The problem of a relative unavailability of ICU capacity during future COVID-19 waves can be solved by using a minimalist TAVI [23] or nurse-led TAVI approach with only local anaesthesia [11].

## Conclusion

In conclusion, we have demonstrated that the case mix of patients undergoing TAVI during the COVID-19 pandemic did not differ substantially from that before this crisis. While the number of TAVI procedures in both cohorts was nearly the same, a significantly lower percentage of patients underwent the TAVI procedure under general anaesthesia, with more transaxillary and fewer transapical TAVIs compared to the situation pre-COVID-19. The outcomes in terms of complications and mortality after TAVI during the COVID-19 pandemic are comparable to those in the pre-COVID-19 era.

## Supplementary Information

**Table S1.** 5‑year volume of Dutch heart centres performing TAVI procedures [21]
