# Supplementary material for: Transcatheter aortic valve implantation amid the COVID-19 pandemic: a nationwide analysis of the first COVID-19 wave in the Netherlands
Source: Neth Heart J. 2022 Jun 1;30(11):503–9. doi: 10.1007/s12471-022-01704-9 (PMC9158307; doi:10.1007/s12471-022-01704-9)
Supplement: Supplementary file 1 — Table S1. 5‑year volume of Dutch heart centres performing TAVI procedures [21] [file 12471_2022_1704_MOESM1_ESM.docx]

**Table S1.** 5-year volume of Dutch heart centres performing TAVI procedures (21)

| TAVI centre | Number of TAVI procedures 2016 – 2020 |
| --- | --- |
| AUMC | 1230 |
| Amphia | 797 |
| St. Antonius | 947 |
| Catharina | 971 |
| Erasmus | 939 |
| Haga | 460 |
| Isala | 693 |
| MCL | 276 |
| MST | 644 |
| MUMC | 717 |
| OLVG | 436 |
| Radboud | 810 |
| UMCG | 651 |
| UMCU | 700 |

AUMC = Amsterdam University Medical Centre, MCL = Medical Centre Leeuwarden, MST = Medisch Spectrum Twente, MUMC = Maastricht University Medical Centre, OLVG = Onze Lieve Vrouwe Gasthuis, TAVI = transcatheter aortic valve implantation, UMCG = University Medical Centre Groningen, UMCU = University Medical Centre Utrecht.
